# Area-level deprivation and geographic factors influencing utilisation of General Practitioner services

**DOI:** 10.1016/j.ssmph.2021.100870

**Published:** 2021-07-11

**Authors:** Peter Barlow, Gretta Mohan, Anne Nolan, Seán Lyons

**Affiliations:** a Economic and Social Research Institute, Whitaker Square, Sir John Rogerson's Quay, Dublin 2, Dublin, Ireland; b School of Economics, Trinity College, Dublin, Ireland

**Keywords:** General practitioner (GP) services, Primary care, GP utilisation, Healthcare supply, Geographic access, Ireland, Healthcare utilisation, Area-level deprivation

## Abstract

Inequities in access to General Practitioner (GP) services are a key policy concern given the role of GPs as gatekeepers to secondary care services. Geographic or area-level factors, including local deprivation and supply of healthcare providers, are important elements of access. In considering how area-level deprivation relates to GP utilisation, two potentially opposing factors may be important. The supply of healthcare services tends to be lower in areas of higher deprivation. However, poorer health status among individuals in deprived areas suggests greater need for healthcare. To explore the relationship of area-level deprivation to healthcare utilisation, we use data from the *Healthy Ireland* survey, which provided a sample of 6326 respondents to face-to-face interviews.

A u-shaped relationship between GP supply and area-level deprivation is observed in the data. Modelling reveals that residing in more deprived communities has a strong, statistically significant positive association with having seen a GP within the last four weeks, controlling for individual characteristics and GP supply. All else equal, residing in an area ranked in the most deprived quintile increases the odds of a respondent having visited the GP in four weeks by 1.43 (95% Confidence Interval: 1.15–1.78), compared to the least deprived quintile (p-value< 0.001). The findings indicate that the level of deprivation in an area may be relevant to decisions about how to allocate primary care resources.

## Highlights

• GP utilisation is higher amongst those in more deprived areas.
• GP supply is lower in middle income areas compared to deprived or affluent areas.
• Differences in GP supply and GP utilisation occur across deprivation quintiles.
• Other geographic factors are not found to be significant in determining GP utilisation.

## 1 Introduction

Equity of access is a core tenet of healthcare policy in countries such as Ireland and the factors which affect it warrant considerable scrutiny. Geographic or area-level factors, such as the supply of healthcare providers and levels of deprivation, can have important effects on utilisation of services. In Ireland, as in many other countries, General Practitioners (GPs) act as gatekeepers for secondary care services, so potential inequities in access to GP services are a key policy concern. Access to primary care has also been emphasised on the international health policymaking stage as a priority for national governments. The World Health Organization (WHO) argues that universal healthcare coverage and the health-related sustainable development goals can only be achieved through a stronger emphasis on primary care (WHO, 2019). The Organisation of Economic Co-operation and Development (Organisationof Economic Cooperation and Development, 2018) espouse the view that primary care has the “potential to improve health, reduce socioeconomic inequalities in health, and make health care systems people-centred”.

The Irish government's *Sláintecare Action Plan,* a strategy for healthcare reform, prioritises developing primary and community services so that “everyone will have entitlement to a comprehensive range of primary, acute and social care services” (Department of Health, 2019a, p. 8). Despite a recognition among policymakers of the importance of access to primary care in Ireland, until recently there had been an absence of policy interventions to encourage GPs to locate in underserved or deprived areas. Smith et al. (2019) presents evidence of an undersupply of GPs in areas that had experienced high population growth in recent years relative to the rest of the country. However, a newly adopted contact between the Irish government and GPs has arranged for the allocation of €2 million to GPs who locate in deprived areas (Health Service Executive, 2019). We note that in the UK, bursaries of £20,000 were offered to incentivise GPs to locate in communities which experienced recruitment shortages (NHS England, 2016), typically deprived areas. Barr et al. (2014) study the impacts of resource allocation to deprived areas in NHS England on mortality, in the context of proposed changes to the funding formula applied to local areas, concluding that where policies which provide additional resources to deprived localities are dropped this may widen health inequalities.

Notwithstanding the consideration of area-level deprivation in the development of health policy, the relationship between area-level deprivation and GP utilisation is relatively unexplored in academic literature. The theoretical framework concerning healthcare utilisation formulated by Aday and Andersen (1974, 1981) provides the basis for the consideration of access to healthcare in this paper. The factors under investigation - the level of deprivation in one's residential area and the level of spatial access to GPs in one's area – are regarded as ‘enabling factors’ in the Andersen framework of healthcare utilisation. This implies that they affect utilisation through their impact on access to primary care services.

Mooney (1983) argued that the supply of healthcare services is an important determinant of access. In general, the supply of healthcare resources has been shown to be inversely correlated with area-level deprivation (i.e., the ‘inverse care law’) (Hart, 1971). Utilisation arises from the interaction of supply from the provider and demand from the patient. Therefore, the impact of area-level deprivation on utilisation depends on the relative strength of the potential downward pressure on supply of GP care from the inverse care law, and the upward pressure on demand from increased healthcare need in deprived areas. This investigation seeks to determine the comparative strength of the two forces acting on GP utilisation in the study setting of Ireland.

The remainder of this paper is structured as follows. The next section explains the organisation of the Irish healthcare system. Literature concerning geographic factors which affect access and utilisation is then outlined. The methods employed to identify the effect of spatial variables and area-level deprivation on GP utilisation in Ireland are described. The results are presented, discussed and the conclusions from the analysis are summarized in the final section.

## 2 Institutional context

Ireland's healthcare system relies on a mixture of public and private provision. Typically, GPs are a patient's first point of contact with the system, acting as gatekeepers to specialist care that is often provided in public hospitals. Unlike other European countries, Ireland does not provide universal public access to primary care. A two-tier system characterises patients as category 1, public patients, who are entitled to a medical card under the General Medical Services (GMS) scheme, or category 2, private patients.

Category 1 patients hold a medical card that entitles them to free consultations with a GP with whom they are registered. The GP is reimbursed by capitation for the provision of care. Medical cards are provided to applicants with low incomes or illnesses that could result in significant financial hardship if they had to pay for care. Medical card holders are subject to small co-payments for prescribed medicines and entitled to free care in public hospitals. In 2018, 33% of the Irish population held a medical card (Department of Health, 2019b). A further 10% held a GP visit card, which provides free GP consultations for otherwise private patients. Qualification for a GP visit card is on the basis of a slightly higher income threshold, although those aged over 70 years, under 6 and carers are automatically entitled.

Category 2 patients pay the full market price for GP services at point of use, with the average visit costing €52.50 (Connolly et al., 2018). They also pay the full cost of medicines subject to a monthly deductible. They are entitled to free or subsidised care in public hospitals, with co-payments for Emergency Department (ED) attendances and in-patient nights.

Approximately 43% of the population purchase private health insurance (Department of Health, 2019b), which typically provides faster access to elective hospital care. Some private plans provide limited coverage of primary care expenses, mainly via partial refunds. Medical cardholders (public patients) can avail of private health insurance.

GPs in Ireland are privately operated, self-employed agents. There are no restrictions on where a GP can locate, but up until 2012, there were constraints on the location of GPs with GMS contracts for medical card patients.

## 3 Review of literature

In a paper concerned with the socio-organization of healthcare resources, Donabedian (1972) contends that socio-economic factors have an important effect on an individual's access to healthcare. Hart (1971) lists numerous factors that present obstacles to accessing primary care in deprived communities in the UK. Issues range from difficulties in recruiting staff, poor building quality and longer patient lists. In a systematic literature search of papers on transportation barriers to healthcare, Syed et al. (2013) notes that vulnerable communities like those on low incomes are particularly affected by transportation barriers to healthcare. The combination of factors pertaining to deprived areas identified by Hart (1971) and Syed et al. (2013) suggests that residents of deprived areas may, by virtue of residing in these areas, have problems accessing primary care.

These obstacles to access may manifest themselves as poorer quality facilities, longer waiting times and greater strain on services. Using the theoretical underpinnings of the inverse care law, Mercer and Watt (2007) demonstrated that patients in deprived areas of Scotland generally take longer to access care and are less satisfied with access. The study employs a 6-item patient enablement instrument to analyse access to care in areas with different unemployment rates to identify the association between deprivation and access. How long patients wait for their clinical encounters, the timeliness of appointment, time spent with the doctor, overall satisfaction and whether the respondent would recommend their doctor are rated. In a similar study, McLean et al. (2006), finds that although there was no systematic link between GP service quality and socioeconomic deprivation, 17 of the 33 indicators used as measures of quality are negatively associated with deprivation. A lack of access to healthcare in deprived areas has also been recognised in the United States; using data from the Medical Expenditure Panel Survey (MEPS), Kirby and Kaneda (2005), demonstrate that those in deprived areas are less likely to have a usual source of care and receive recommended preventative medicine.

The importance of deprivation in the consideration of access depends not only on its direct impact on healthcare demand and supply but also on how individuals perceive relative access in their area. Comber et al. (2011) finds that individual socio-economic disadvantage and greater geographic distance to GP and hospital services have negative impacts on public perceptions of access to healthcare. This complex relationship between deprivation, geographic factors and healthcare utilisation is further affirmed by Field and Briggs (2001) in an examination of GP utilisation in Northampton, UK. They find that the impact of distance to the GP is mediated by socio-economic factors and suggest that those furthest from the GP had better access to a car while those at an intermediate distance from the GP rely on public transport.

Demand for healthcare has also been found to be higher in deprived areas. Carlisle et al. (2002) finds that there were 44% more out of hours contacts in more deprived areas of Nottinghamshire, UK. In Canada, neighbourhoods with a significantly higher number of low-income households are more likely to have higher levels of healthcare utilisation and poorer health outcomes (Lemstra et al., 2006). Using the UK Practical Research Datalink in conjunction with the Index of Multiple Deprivation (IMD) from 2010, Charlton et al. (2013) find that multimorbidity is higher in deprived areas, with implications for healthcare utilisation in these areas. The study finds that higher costs of healthcare use are associated with increasing deprivation and morbidity.

Greater utilisation of primary care in deprived areas may be in part attributable to poorer health status in these areas. The landmark study of the Marmot Review into health inequalities in England (Marmot et al., 2010a, 2010b) highlighted the existence of a social gradient in health outcomes, presenting evidence that the most economically disadvantaged neighbourhoods had substanitally poorer life expectancy and greater disability levels compared to more affluent neighbourhoods. This Marmot study was revisted a decade later (Marmot, 2020), finding that the health gap had grown between wealthy and deprived areas of England in the ten year period, concluding that place of residence matters for one's health. Diez Roux and Mair, (2010) provides an overview of recent studies concerned with neighbourhood and health, concluding that there is substantial evidence that health is spatially patterned according to social patterning of residential environments. Previously, Oakes (2004, p.1929) had noted that epidemiologists have long recognised that people living in different neighbourhoods have different outcomes, proposing that “spatial variation in morbidity and mortality is somehow associated with the clustering of genetic predispositions, cultural norms, opportunity structures, and/or environmental conditions”. Moreover, Ross and Mirowsky (2001) find that disorder arising from living in deprived areas leads to poorer health outcomes. Stafford and Marmot (2003) test the independent effects of both individual and area-level deprivation in a study of 10,000 civil servants in the UK. The study further examines two models concerning whether the cause of the area-level effect is socio-economic inequality or collective resources. They find that both individual and area-level deprivation affect health outcomes and conclude that the results of the analysis are consistent with an explanation rooted in greater reliance on more limited collective resources in deprived areas.

There is also a wide literature describing the relationship between area-level deprivation and specific health conditions (see Diez Roux and Mair (2010)). In Germany, analysis of a nationwide dataset in conjunction with the German Index of Multiple Deprivation shows higher prevalence of diabetes in more deprived communities (Grundmann et al., 2014). In a study of 200 neighbourhoods of Australia, Brennan and Turrell (2012) find the prevalence of arthritis to be significantly greater in socially disadvantaged neighbourhoods, independent of individual-level factors. A US-based study finds that better neighbourhood conditions such as walkability, safety, social cohesion and availability of healthy foods were associated with lower hypertension of residents, though the effect was attenuated or disappeared when race/ethnicity was accounted for (Mujahid et al., 2008). Kawachi and Berkman (2003) outlines a comprehensive relationship between neighbourhood deprivation and health outcomes such as infectious disease, infant health and asthma. A systematic review of multilevel studies relating to child and adolescent health in deprived neighbourhoods found that on average 10% of variation on health outcomes was explained by neighbourhood factors (Sellström & Bremberg, 2006).

Hitherto, the importance of area-level deprivation in influencing the utilisation of healthcare services in the Irish context has been relatively unexplored. In mapping the provision of GPs in Ireland, Teljeur et al. (2010) finds no obvious inequity in the travel times to the nearest GP for residents of deprived areas compared to the rest of the Irish population. Recently, a study by Smith et al. (2019) reveals that GP supply is lowest in areas of high population growth, implying that GP supply might not have kept up fully with increasing demand.

Smith (2007) uses data from four Irish teaching hospitals around Dublin, finding evidence of increased ED utilisation in areas without a good supply of primary care services. The study also finds that the hospital catchment with the highest level of deprivation has a higher proportion of urgent cases, and is characterised by greater odds of self-discharging. In another relevant investigation, Sexton and Bedford (2016) report that areas with low GP supply and high deprivation have higher rates of ED admission in a study of inpatient discharge data in Ireland.

The investigation undertaken by this paper aims to add to literature on the relationship between area-level deprivation and an individual's use of primary healthcare, accounting for the individual's material and health circumstances as well as the local supply of GPs.

## 4 Data and methods

### 4.1 Data

The *Healthy Ireland* (HI) survey began in 2015 as an annual cross-sectional survey designed to be representative of residents of the Republic of Ireland above the age of fifteen years (Department of Health, 2016). The purpose of the survey is to capture a picture of the health of the population. The data is collected by a private company, Ipsos MRBI, on behalf of the Department of Health. The research team submitted an application to the Department of Health in Ireland for use of the HI data. The 2016 wave of HI is used in this analysis since it corresponds with data on GPs located in Ireland in 2016. A multi-stage sampling design was used to select a sample of residents across the country, fully described in Ipsos MRBI (2017). The initial stage of the sampling process involved selecting a representative distribution of sampling points across Ireland, where all electoral divisions were stratified by region and socio-demographic factors (Department of Health, 2017). Then within the electoral divisions which provided 686 sampling points, the An Post Geodirectory, which contains all addresses in Ireland, was used to select specific addresses to be contacted for interview. A random start point and systematic skip was employed to select twenty addresses in each sampling point, where each of these addresses were visited by an interviewer. In each household, the interviewer randomly selected one individual above the age of fifteen years for sampling. Between September 2015 and May 2016, 7498 respondents were interviewed, where the realised survey response rate was 59.9% (Ipsos MRBI, 2017). Of these respondents, 6326 provided complete responses to questions used in this analysis from an anonymised microdata file. A comparison of the characteristics of the full surveyed sample and those used for analysis are included in Table A1 of the Supplementary File, where we note that both samples were broadly similar in composition.

The association between the characteristics of the area of residence of HI survey participants and their utilisation of GP services is examined. A multiple deprivation indicator, the Haas Pratschke (HP) index, is used as a proxy for the level of deprivation, and the extremity of deprivation or affluence in a small area (Haase & Pratschke, 2016).

### GP data

A list of GPs in Ireland was compiled originally for a 2010 study (Teljeur et al., 2010), informed by records from the Irish College of General Practitioners (ICGP) and the Irish Medical Directory, which was updated for 2016.^1^ The location of GPs and HI participants were mapped using geographical information system (GIS) techniques, specifically using QGIS software.

### 4.2 Outcome of interest

The HI survey enquired as to a respondent's contact with GP services in two parts. The respondent was first asked whether they had attended a GP in the previous 12 months, to which the respondent could give a ‘yes’ or ‘no’ repsonse. Where the respondent had answered ‘yes’, a follow up question asked how often the respondent attended the GP in the previous 4 weeks, to which the respondent could report the number of visits to the GP. For the purposes of modelling, a binary outcome variable was created for the analysis; the outcome variable ‘Visited GP in previous 4 weeks’ took a value of 1 where the respondent had reported 1 or more visits to the GP in the previous 4 weeks, and 0 where the respondent had reported zero visits to a GP in the previous 4 weeks.

We note that data collection for this survey occurred in the months between September 2015 and May 2016, and thus responses relating to use of GP services in the previous four weeks reflect attendances in the seasons of Autumn, Winter and Spring. Unfortunately, information on the precise day or week of the respondent's interview was not available to the research team, and therefore possible seasonal effects could not be taken into account.

### 4.3 Associations of interest

#### Area-level deprivation

The level of deprivation in the residential area of the HI respondent is proxied by the HP deprivation index. This index is compiled from measures of the demographic profile, social class composition and labour market conditions of 18,488 small areas across Ireland. Administrative statistics such as the number of single parent households and the unemployment rate inform the index (further details on the composition of the HP index is provided in the Supplementary File). The HP index is comparable with international indices of multiple deprivation, such as those employed in the UK (Noble et al., 2006). We also note that access to services, including healthcare itself, has been considered as a domain within UK indices of multiple deprivation, but it is not included in the HP index.

For the purposes of analysis, scores on the HP index were aggregated into quintiles – where quintile 1 represents the most deprived. The association between area-level deprivation and the use of GP services is likely to be affected by two potentially opposing factors. Evidence demonstrates that area-level deprivation may have a negative effect on access, and by extension utilisation (Carlisle et al., 2002). However, because individuals from deprived areas are more likely to have poorer health, there is likely to be higher demand and higher utilisation. Therefore, the direction of effect of area-level deprivation on utilisation depends on the respective sizes of the two forces influencing it. Our analysis endeavours to measure the net effect of these factors. This variable allows us to test the following hypothesis:

Hypotheis 1 : An individual in an area of greater area-level deprivation is more likely to visit the GP in the previous 4 weeks.

#### GP supply variable

The mapping of the location of GPs and HI participants afforded the creation of a ‘geographic supply’ variable. As outlined in previous research (Mooney, 1983), the supply of healthcare services itself may influence access and utilisation of health services, and thus should be controlled for in analyses where possible. This has been further substantiated by research from Switzerland and Sweden which has indicated that increased supply of GPs in a person's area increases their rate of GP and healthcare utilisation (Beckman & Anell, 2013; Busato & Künzi, 2008). An indicator of the degree of GP spatial access was generated using QGIS software, establishing the number of GPs within a 1.6 km circular radius of the HI respondent's residence (estimated to be a 20-min walking distance). For some HI respondents there were no GPs within 1.6 km, and then for those with a GP in walking distance, the extent of spatial access was split into quintiles. A greater level of spatial access may facilitate greater utilisation of GP services, as well as greater satisfaction (Schmittdiel et al., 1997), ease of making an appointment, etc. Thus, we hypothesise that a more extensive spatial access of GPs will be associated with higher utilisation. In this paper, we test the following hypothesis based on the relationship between geographic supply and utilisation of healthcare services:

Hypothesis 2 The spatial access to GPs in an individual's residential area is positively associated with greater GP visitation in the previous 4 weeks.

To test the robustness of the findings of this paper, an analysis of two further variables indicative of GP supply is conducted, namely distance to the nearest GP and a measure of the workload of the nearest GP (described in Mohan et al., 2019)). Distance to the nearest GP was determined by assessing the road distance from an individual's address to their nearest GP based on data from Open Street Maps. The variable which proxies for GP workload in the supplementary file is estimated by determining the number of individuals whose closest GP was also the respondent's closest GP. Results from estimation of models using all of the proxies for GP supply can be found in the supplementary file in Tables A2 A3 and A4.

#### Other covariates

We also include variables which indicate an individual's demographic, socio-economic and health status. These covariates allow us to discern the impact of individual demographic and health circumstances which can be disentangled from the effect of residing in a deprived area on GP utilisation.

### 4.4 Model

To estimate the impact of area-level deprivation and the degree of spatial access to GPs in one's locality on whether an individual had ‘visited the GP in the previous 4 weeks’, a logistic regression model is employed. Three iterations of the model assessing the impact of area-level deprivation on GP utilisation are represented below as:

(1) $$Pr\left(u_{i}=\;1\right)=\;\frac{\text{exp}\left(\alpha \;+\;\beta_{1}Dep_{i},\;+\;\beta_{2}X_{i}\right)}{1\;+\;\text{exp}\left(\alpha +\;\beta_{1}Dep_{i},\;+\;\beta_{2}X_{i}\right)}$$

(2) $$Pr\left(u_{i}=\;1\right)=\;\frac{\text{exp}\left(\alpha +\;\beta_{1}Dep_{i}+\;\beta_{2}X_{i}+\;\beta_{3}D_{i},\right)}{1\;+\;\text{exp}\left(\alpha +\;\beta_{1}Dep_{i}\;+\;\beta_{2}X_{i}+\;\beta_{3}D_{i}\right)}$$

(3) $$Pr\left(u_{i}=\;1\right)=\;\frac{\text{exp}\left(\alpha +\;\beta_{1}Dep_{i}+\;\beta_{2}X_{i}+\;\beta_{3}D_{i}+\;\beta_{4}S_{i}\right)}{1\;+\;\text{exp}\left(\alpha +\;\beta_{1}Dep_{i}+\;\beta_{2}X_{i}+\;\beta_{3}D_{i}+\;\beta_{4}S_{i}\right)}$$

**Fig. 1:**
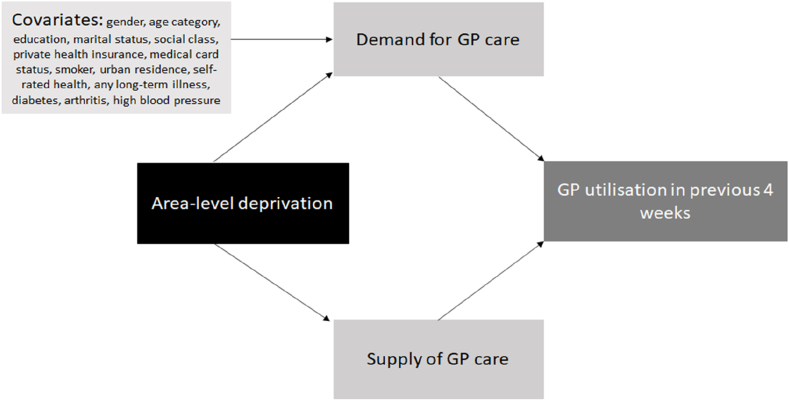
Diagrammatic description of relationships studied: arrows represent the effects assessed.

Where $u_{i}$ denotes the dependent variable, the utilisation of GP services in the previous 4 weeks by individual i. The function, f(), includes α, a constant term; $\;\beta_{1}$, the main parameter to be estimated, which represents the influence of area-level deprivation on GP utilisation as captured by the variable $Dep_{i}$, the HP index quintile of deprivation for the area in which the individual resides. The expression *exp* indicates a value raised to the power of the value indicated. For model 1, the additional included covariates of the individuals age and gender are denoted as $X_{i}$, the influence of which are estimated as$\;\beta_{2}$. Model 2 includes all the variables of model 1, as well as a vector of covariates denoted, $D_{i}$, which may influence an individual's healthcare demand including medical card status, private health insurance status, marital status, whether the person may be categorised into an unskilled social class, level of education, whether they smoke, whether they had an illness in past 12 months, whether they have specific health conditions which may be managed in the primary care setting including diabetes, arthritis and high blood pressure, as well as whether they live in an urban area and the region of the country. Model 3 includes the variables of model 2 and an independent variable which captures the supply of GPs, $S_{i}$ as measured by the number of GPs within a 1.6 km radius. The data analysis for this paper was carried out using STATA 16.1. A diagramtic description of the relationships studied in this paper is illustrated in Fig. 1.

#### Sensitivity analysis

A number of sensitivity analyses are conducted which employ different approaches to analyse the association between area-level deprivation and GP utilisation to assess the robustness of the main model results. These included a logistic regression of whether an individual had attended a GP in the previous 12 months, the results of which are displayed in Supplementary File Table A5. A linear regression on the number of visits to the GP in the previous month is also reported in Supplementary File Table A6.

### Odds ratios

The results in this paper are presented as odds ratio. A statistically significant odds ratio greater than 1 indicates that the independent variable examined is associated with a higher likelihood of the outcome occurring, while an odds ratio less than 1 indicates a lower probability (Szumilas, 2010).

## 5 Results

**Fig. 2:**
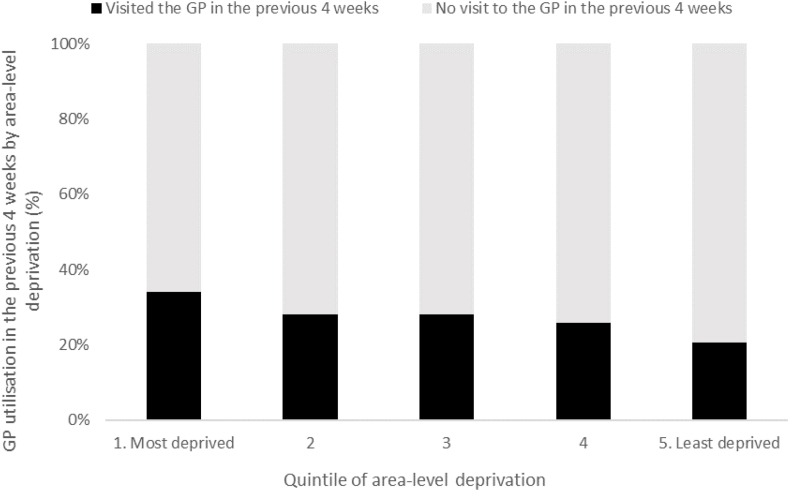
Visited GP in previous 4 weeks by quintile of area-level deprivation.

Table 1 provides the summary statistics of the analytical sample, where over a quarter (27.4%) of respondents had reported attending the GP in the previous 4 weeks. Fig. 2 shows the proportion of sample respondents who had visited the GP in the previous 4 weeks for each quintile of area-level deprivation. Those who resided in the most deprived areas were most likely to have attended the GP in the previous 4 weeks, with a gradient across levels of deprivation.

**Table 1:** Summary statistics for the HI sample.

Characteristic	Category	Percent
*GP attendance*	Attended the GP in the previous 4 weeks	27.4
	Not attended the GP in the previous 4 weeks	72.6
*Area-level deprivation*	Quintile 1 (Most deprived)	19.2
	Quintile 2	22.3
	Quintile 3	20.1
	Quintile 4	20.6
	Quintile 5 (Least deprived)	17.7
*Gender*	Male	46.4
	Female	53.6
*Age class*	15–24	7.5
	25–44	33.8
	45–64	32.9
	65 or greater	25.8
*Education*	Primary	10.3
	Secondary	46.5
	Tertiary	43.2
*Marital status*	Married	56.2
	Not married	43.8
*Social class (Manual labourer)*	Yes	14.3
	No	85.7
*Smoker*	Yes	16.0
	No	84.0
*Private health insurance status*	Insured	51.1
	Uninsured	48.9
*Medical card status*	No medical card	58.1
	GP visit card	6.4
	Medical card	35.5
*Region*	Dublin	22.2
	Non-Dublin Leinster	26.4
	Munster	28.9
	Connaught/Ulster	22.4
*Urban*	Urban	61.0
	Rural	39.0
*Self-rated health*	Good or very good	72.1
	Fair, poor or very poor	27.9
*Long term illness (past* 12 months*)*	Yes	29.7
	No	70.3
*Diabetes*	Yes	4.8
	No	95.2
*Arthritis*	Yes	12.3
	No	87.7
*High blood pressure*	Yes	15.5
	No	84.5
*GP supply: Number of GP within 1.*6 km *(walking distance)*	Zero GPs in walking distance	36.6
	Quintile 1 (Least GPs)	15.0
	Quintile 2	12.1
	Quintile 3	11.8
	Quintile 4	13.2
	Quintile 5 (Most GPs)	11.3
*Number of observations*		6326

**Fig. 3:**
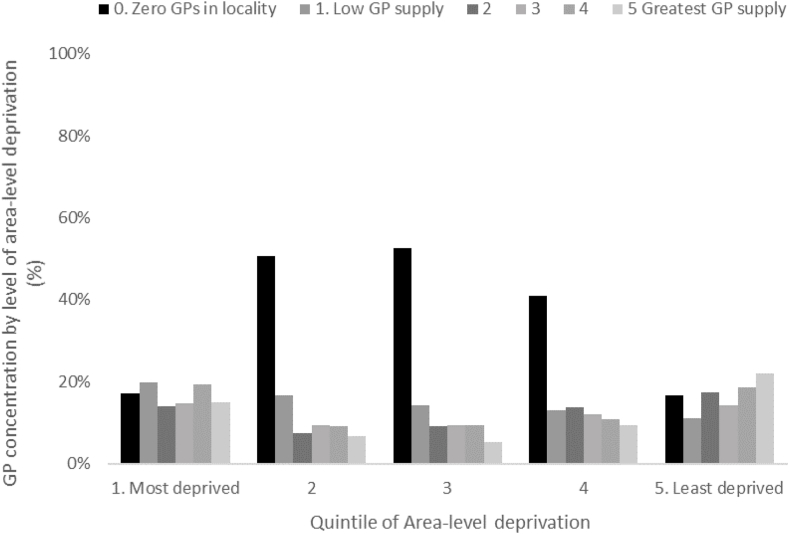
Percentage of individuals in a GP spatial access quintile by area-level deprivation quintile.

Fig. 3 plots the supply of GPs (as measured by the density of GPs within a 1.6 km radius) for each of the quintiles of area-level deprivation. A u-shaped relationship between the supply of GPs and area-level deprivation was apparent, where the heaviest concentrations of GPs were in the most deprived and least deprived areas. For moderate levels of deprivation (quintiles 2, 3 and 4) there was a relatively lower supply of GPs.

The estimated odds ratios associated with area-level deprivation on utilisation of GP services are presented in Table 2. The basic model, model 1, which includes the deprivation quintiles, age and gender, estimates that the effect of residing in a deprived area, relative to an affluent area, is both statistically significant and large in relation to whether an individual visited a GP in the previous 4 weeks. The estimated effect was attenuated with further adjustment for socioeconomic, health and supply-side variables in the full model specification, model 3. All else equal, residing in an area that was ranked in the most deprived quintile increased the odds of a respondent having had contact with a GP in the previous 4 weeks by 1.43 (95% Confidence Interval: 1.15–1.78), compared to the most affluent quintile (p < 0.001). No effect was observed from the proxy variable for GP supply in the analysis described in Table 2. Tables A2, A3 and A4 also indicate that GP supply had a negligible impact on GP utilisation based on three proxies of GP supply, namely GP concentration, distance to GP and GP workload.

**Table 2:** Logistic regression results for GP attendance in the previous 4 weeks, presented as odds ratios.

**GP visit in previous 4 weeks**	**Reference category**	**Basic model**	**Full model without GP supply**	**Full model including GP supply**
Model		(1)	(2)	(3)
Deprivation quintile 1 (Most deprived)	Least deprived quintile	1.773*** (0.172) [1.466–2.145]	1.402** (0.155) [1.129–1.741]	1.430** (0.159) [1.149–1.779]
Deprivation quintile 2	Least deprived quintile	1.343** (0.130) [1.111–1.623]	**1.240** (0.137) [0.998–1.541]	1.260* (0.140) [1.014–1.567]
Deprivation quintile 3	Least deprived quintile	1.413*** (0.139) [1.165–1.713]	1.324* (0.146) [1.068–1.643]	1.342** (0.148) [1.081–1.667]
Deprivation quintile 4	Least deprived quintile	1.224* (0.121) [1.008–1.486]	1.247* (0.134) [1.011–1.539]	1.255* (0.135) [1.016–1.549]
Male	Female	0.722*** (0.042)	0.726*** (0.045)	0.725*** (0.045)
Age 25-44	Age 18-24	0.968 (0.122)	0.870 (0.120)	0.867 (0.120)
Age 45-64	Age 18-24	1.295* (0.162)	0.909 (0.125)	0.910 (0.125)
Age 65+	Age 18-24	2.724*** (0.340)	1.017 (0.148)	1.022 (0.149)
Secondary educated	Primary educated		0.928 (0.099)	0.928 (0.100)
Tertiary educated	Primary educated		1.005 (0.122)	1.004 (0.122)
Married	Unmarried		1.057 (0.070)	1.056 (0.0704)
Manual labourer	Other profession		0.945 (0.0837)	0.943 (0.0836)
Smoker	Non-smoker		0.975 (0.0844)	0.976 (0.0843)
Private health insurance	No private health insurance		1.156 (0.088)	1.160 (0.0881)
GP visit card holder	No medical card		1.646*** (0.204)	1.649*** (0.206)
Medical card holder	No medical card		2.095*** (0.174)	2.100*** (0.175)
Region: Non-Dublin Leinster	Region:Dublin		0.820* (0.080)	0.794* (0.0850)
Region: Munster	Region:Dublin		0.851 (0.08)	0.814* (0.083)
Region: Connaught/Ulster	Region:Dublin		0.889 (0.092)	0.845 (0.0963)
Urban	Rural		1.002 (0.073)	0.987 (0.100)
Long term illness	No long term illness		1.929*** (0.144)	1.920*** (0.144)
Good or better self-rated health	Fair, poor or very bad self rated health		0.498*** (0.0434)	0.496*** (0.0433)
Arthritis	No arthritis		1.237* (0.117)	1.241* (0.118)
Diabetes	No diabetes		1.070 (0.149)	1.068 (0.149)
High blood pressure	No high blood pressure		1.408*** (0.119)	1.415*** (0.120)
GP concentration quintile 1 (Lowest supply of GPs in locality)	Zero GPs in walking distance			0.980 (0.098) [0.806–1.192]
GP concentration quintile 2	Zero GPs in walking distance			1.212 (0.157) [0.940–1.562]
GP concentration quintile 3	Zero GPs in walking distance			0.960 (0.129) [0.738–1.249]
GP concentration quintile 4	Zero GPs in walking distance			0.871 (0.122) [0.661–1.147]
GP concentration quintile 5 (Most GPs in locality)	Zero GPs in walking distance			1.017 (0.150) [0.761–1.358]
N		6326	6326	6326
Log likelihood		−3563.43	−3307.84	−3303.97
Statistical significance indicated by * p < 0.05 **p < 0.01 ***p < 0.001. Robust standard errors in parentheses. 95% Confidence intervals in square brackets for the main variables of interest (estimates on quintiles of deprivation and GP concentration (supply)). Table A2, A3 and A4 in the Supplementary File provide robustness analysis of other proxies for GP supply, Table A5 and A6 apply other modelling approaches.				

## 6 Discussion

Our results indicate that residing in a deprived area is associated with a higher utilisation of GP services, which may be attributed to factors driving increased demand for GP services in deprived areas. However, the observed factors which affect individual level demand for GP care (e.g. age, sex, health status etc.) do not fully attenuate the effect of area-level deprivation, suggesting that there is a significant residual positive association between area-level deprivation and GP utilisation. This empirical result provides evidence in support of Hypothesis 1, and indicates that there are higher utilisation rates of GPs in deprived areas.

Higher utilisation rates in deprived communities, as demonstrated in this analysis, can impose strain on primary care services in deprived areas (Carlisle et al., 2002). There are several potential mechanisms that might help explain higher healthcare utilisation in deprived areas. We attempt to control for some of these effects through the inclusion of variables related to health status and behavioural characteristics, but unobservable effects from area-level deprivation may still impact upon GP utilisation. We consider four factors which may explain why GP utilisation is higher for residents of deprived areas:

1. **Contagion effect:** Higher morbidity rates in deprived areas (Curtis, 1990) imply that individuals resident in deprived areas may be more likely to have and/or carry illnesses. As a result, individuals may be more likely to live in conditions which increase the probability of infection. Such increased morbidity could exacerbate health inequality (Curtis & Rees Jones, 1998). In the analysis contained in this paper, health status is controlled for, but there may be residual unobservable contagion effects on health within the context of area-level deprivation.
2. **Social effect:** In a study of why socioeconomic disadvantage is correlated with poorer health outcomes, Adler et al. (1994) suggested some activities linked to behaviour may be the root cause of the disparity in health such as diet, drinking and physical activity. Duncan et al. (1993) also outlined that drinking alcohol and unhealthy eating were more prevalent in deprived communities. Less healthy lifestyles, concentrated in deprived neighbourhoods, may result in pockets of poorer health (Diez Roux and Mair, 2010), increasing the need for GP utilisation. Whether an individual is a smoker is controlled for in the analysis, which provides a proxy for whether that respondent engages in unhealthy behaviours. Being a smoker was not associated with GP visitation in the previous 4 weeks (Table 2), though wider behavioural characteristics were unobserved in this analysis. There is a large literature explaining the impacts of wider social factors on an individual's health (Braveman, 2003; Braveman et al., 2011, Braveman and Gottlieb, 2014; Marmot et al., 2010a, 2010b; Marmot, 2020; World Health Organization, 2019). A review of systematic reviews of the wider determinants of health concludes that this area merits greater research and significant policy intervention (Bambra et al., 2010).
3. **Resource/Poverty effect:** Health outcomes in deprived areas may be poorer because these areas depend more on collective resources, both material and social resources, such as public services and social supports. Stafford and Marmot (2003) found that the health inequality between deprived and affluent areas could be explained by the fact that deprived areas had fewer collective resources such as area-level amenities, services, job opportunities and social supports. Concentrations of wealthier individuals in certain areas may be better able to attract amenities and social supports to those areas which can support the health system. Individuals in poorer areas without these may be more reliant on existing GP and primary healthcare services.
4. **Environmental effects:** Areas of lower deprivation may be characterised by environments that are more amenable to better health outcomes. Neighbourhoods with lower levels of area-level deprivation have been found to be more ordered, safer and less stressful (Ross & Mirowsky, 2001), conducive to better health. It is also argued that less deprived communities have a better built environment, quality of housing and access to food.

Previous research suggests a greater supply of healthcare services may be associated with higher utilisation. However, we found no evidence of this for GPs in Ireland. This may partly be explained by the u-shaped relationship between supply and area-level deprivation observed in Fig. 2. Three explanations are offered to describe this u-shaped relationship:

1. Higher GP supply in the most deprived areas may be attributed to the incentives provided by the GMS medical card payment system. Where a GP has a patient with a medical card, a guaranteed capitation fee is provided to that GP regardless of patient visitation rates. Additional allowances for sick leave, maternity and study leave, and grants to support the premises, hiring secretarial staff and nursing staff (which depend on the number of medical card patients on their GMS list) are also available (Competition Authority, 2010). Because of socio-economic disadvantage and lower incomes, more deprived areas are more likely to be characterised by higher concentrations of individuals entitled to medical cards, which may create an incentive for GPs to locate there.
2. A lower relative supply of GPs in areas of moderate deprivation may be attributable to higher population growth in these areas in recent years. This has not been met with a similar growth in the provision of GP services (Smith et al., 2019). As a result, these areas have been found to have lower GP provision per capita than more established areas. These high growth areas are typically middle income areas which lie outside cities. This may suggest an unfulfilled need for GPs in middle income areas.
3. The higher concentration of GPs in affluent areas may be explained by the attraction of GPs to more prosperous catchments characterised by wealthier patients who can afford private fees (consistent with the inverse care law). This is consistent with the findings of Mercer and Watt (2007) and McLean et al. (2006).

The potential for an undersupply of GP care in very deprived areas associated with the inverse care law may have been avoided in Ireland. For areas of moderate deprivation/affluence the concentration of GPs was relatively lower, implying that there may be an undersupply in these areas which may have ramifications for quality of care and time with patients. Nevertheless, we found no evidence that such variations in supply led to differences in utilisation.

The estimated effect of a number of other variables included in the models reported in Table 2 are also noteworthy. The variable with the strongest association with having visited a GP in the previous 4 weeks was whether the individual possessed a medical card (i.e. had free consultations). Those who possessed a medical card were twice as likely to have visited the GP in the previous 4 weeks as those who had no medical card. We note that medical cards are disproportionately held in more deprived areas, as evidenced in Supplementary File Figure A3. Similarly, holding a GP visit card increased the odds of GP attendances, as did having a long-term illness or high blood pressure. ‘Good’ or ‘better’ self-rated health was associated with a lower odds of attending a GP. We also note that in the final, fully adjusted model, which accounts for GP supply, residing in the regions of Non-Dublin Leinster and Munster was associated with a statistically significant lower odds of GP utilisation, relative to the reference category of Dublin. The reason for this apparent regional influence is unclear and may merit future research investigation.

This study benefits from a large, nationally representative dataset which contains a comprehensive set of demographic, socio-economic and health variables which inform the analysis. However, due to the cross-sectional nature of the data collection, the findings of this investigation point to the association or link between the relationships of interest and it cannot make claims of causality. While the response rate to the survey (59.9%) may be considered good, there remains the potential for some bias from survey non-response, however, the Department of Health *Summary of Findings* report (Department of Health, 2017) and the survey technical report (Ipsos MRBI, 2017) outlines that the respondents were representative of the Irish adult population and thus the results generated from this research should be considered generalisable to the Irish adult population. Longitudinal data from other sources could be used to test this relationship in future studies to derive firmer conclusions. We also note that with the exeption of area of residence, data for the dependent variable and many independent variables are self-reported and thus there is potential for response bias and issues of recall.

## 7 Conclusion

Residing in more deprived areas has a positive and significant association with having had contact with a GP in the previous 4 weeks. In line with Hart (1971), the findings provide evidence of greater demand for healthcare in areas of higher deprivation. However, the distribution of GPs in the context of deprivation in Ireland does not corroborate the theoretical predictions of the inverse care law (Hart, 1971). Rather, a u-shaped relationship between GP supply and area-level deprivation was observed. The evidence presented suggests that there is a discrepancy between supply and utilisation in areas of moderate deprivation, but we found no evidence that such variations in supply affected utilisation of GP services in Ireland.

In conclusion, area-level deprivation appears to have a significant association with an individual's utilisation of GP services, even after controlling for many determinants of individual-level demand for GP care and a proxy for GP supply. This implies that government policy towards development of primary care should consider the extent of deprivation at local level.

## Funding

This research is supported by Ireland’s Health Research Board ‘Inequalities in Access to GP Care in Ireland’ project (HRA-PHR-2014-508). The authors are grateful to the Department of Health in Ireland for access to the *Healthy Ireland* data.

## CRediT Statement

Peter Barlow: Conceptualization, Methodology, Software, Data curation, Investigation, Formal analysis, Writing – original draft, Gretta Mohan: Formal analysis, Investigation, Writing – original draft, Writing- Reviewing and Editing, Project administration, Anne Nolan: Resources, Project administration, Funding acquisition, Investigation, Supervision, Writing- Reviewing and Editing, Seán Lyons: Supervision, Writing- Reviewing and Editing.

## Declaration of competing interests

The authors declare that they have no known competing financial interests or personal relationships that could have appeared to influence the work reported in this paper.

## Footnotes

1 Data on GPs in Ireland in 2016 may be accessed by contact with the corresponding author of Teljeur et al. (2010).
