# Supplementary material for: Area-level deprivation and geographic factors influencing utilisation of General Practitioner services
Source: SSM Popul Health. 2021 Jul 11;15:100870. doi: 10.1016/j.ssmph.2021.100870 (PMC8342788; doi:10.1016/j.ssmph.2021.100870)
Supplement: Multimedia component 1 [file mmc1.docx]

**Area-level deprivation and geographic factors influencing utilisation of General Practitioner services**

**Supplementary file**

1. **Summary Statistics of samples from Healthy Ireland**

**Table A1: Summary statistics for Healthy Ireland full sample and analytical sample used**

| **Variable** | **Category** | | **Full sample**  **(%)** | **Sample used in analysis** |
| --- | --- | --- | --- | --- |
| *GP attendance* | Attended GP in previous 4 weeks | | 29.5 | 27.4 |
|  | Did not attend GP in previous 4 weeks | | 70.5 | 72.6 |
| *Area-level deprivation* | Deprivation quintile 1 (Most deprived)  Deprivation quintile 2  Deprivation quintile 3  Deprivation quintile 4  Deprivation quintile 5 (Least deprived) | | 21.1  22.3  19.6  20.1  16.9 | 19.2  22.3  20.1  20.6  17.7 |
| *Gender* | Male | | 44.3 | 46.4 |
|  | Female | | 55.8 | 53.6 |
| *Age class* | 15-24  25-44  45-64  65 or greater | | 8.1  33.1  32.7  26.1 | 7.5  33.8  32.9  25.8 |
| *Education* | Primary  Secondary  Tertiary | | 11.4  47.3  41.3 | 10.3  46.5  43.2 |
| *Marital status* | Married  Unmarried | | 52.6  47.5 | 56.2  43.8 |
| *Social class (manual labourer)* | Yes  No | | 14.2  85.8 | 14.3  85.7 |
| *Private health insurance status* | Has private health insurance  No private health insurance | | 52.4  47.6 | 51.1  48.9 |
| *Medical card status* | No medical card or GP visit card  GP visit card holder  Medical card holder | | 53.7  6.0  40.4 | 58.1  6.4  35.5 |
| *Smoker* | Yes  No | | 17.3  82.7 | 16.0  84.0 |
| *Urban* | Urban  Rural | | 61.4  38.6 | 61.0  39.0 |
| *Self-rated health* | Good or very good  Fair, poor or very poor | | 81.9  18.0 | 72.1  27.9 |
| *Long term Illness in past 12 months* | Yes  No | | 31.5  68.5 | 29.7  70.3 |
| *Diabetes* | Yes  No | | 5.3  94.7 | 4.8  95.2 |
| *Arthritis* | Yes  No | | 13.1  86.9 | 12.3  87.7 |
| *High blood pressure* | Yes  No | | 15.8  84.2 | 15.5  84.5 |
| *Supply of GPs in locality (quintile)* | 0.No GP in 1.6km  1.Least GPs in 1.6km  2.  3.  4.  5.Most GPs in 1.6km | | 36  15.2  11.9  12  13.4  11.6 | 36.6  15.0  12.1  11.8  13.2  11.3 |
| Sample observations |  | 7,498 | | 6,326 |

1. **Explanation of HP deprivation index**

The deprivation index used in this paper is the Pobal Haas-Pratschke (HP) index of multiple deprivation (Haase and Pratschke 2017). It uses small area datasets to create a multiple deprivation index for the Republic of Ireland. It calculates deprivation scores based on variables which determine demographic profile, social class composition and the labour market situation of the relevant small area. Using Confirmatory Factor Analysis (CFA), the index includes a multitude of variables, the values of which are combined to establish a deprivation valuation for each of the 18,488 small areas in the country (Haase and Pratschke 2017). The variables in each of the domains are outlined as follows:

1. Demographic growth: The Small Area Population (SAP) statistics used to indicate demographic growth are the age dependency rate, population change, proportion of individuals with no or primary education only, proportion of individuals with higher education, number of people per room. This analysis of demographic growth takes account of rural area-level deprivation. Areas of rural decline in Ireland are generally characterised by low population density, reducing population and low levels of education. Therefore, this index takes account of the significant rural population in Ireland and the deprivation therein.
2. Social Class composition: The second group of SAP statistics used by the HP deprivation index provides an indication of social class composition. This includes the proportion of individuals with no or primary education only, the proportion of individuals with tertiary education, the number of individuals per room as well as the proportion of individuals in each social class. This importance of social class is of equal importance to both rural and urban areas. Areas with a traditionally lower social class demographic profile generally have poorer educational attainment, housing and higher unemployment rates.
3. Labour market conditions: The last method by which the HP index takes account of deprivation are the labour market conditions in that area. The labour market situation is an important indicator of urban deprivation. Urban areas with significant unemployment rates generally have significant problems with lower incomes from lack of paid employment. The variables included in this measure are the proportion of lone parents in the area, the proportion of semi and unskilled classes in the area, the male unemployment rate and the female unemployment rate.

Figure A1 provides a visual representation of the process by which the HP index reaches its scores for each small area (Haase and Pratschke 2017 pg. 4). Figure A2 maps the area-level deprivation statistics against area-level income from SAP statistics provided by Ireland’s Central Statistics Office. It finds a relatively strong correlation between these respective measures of disadvantage.


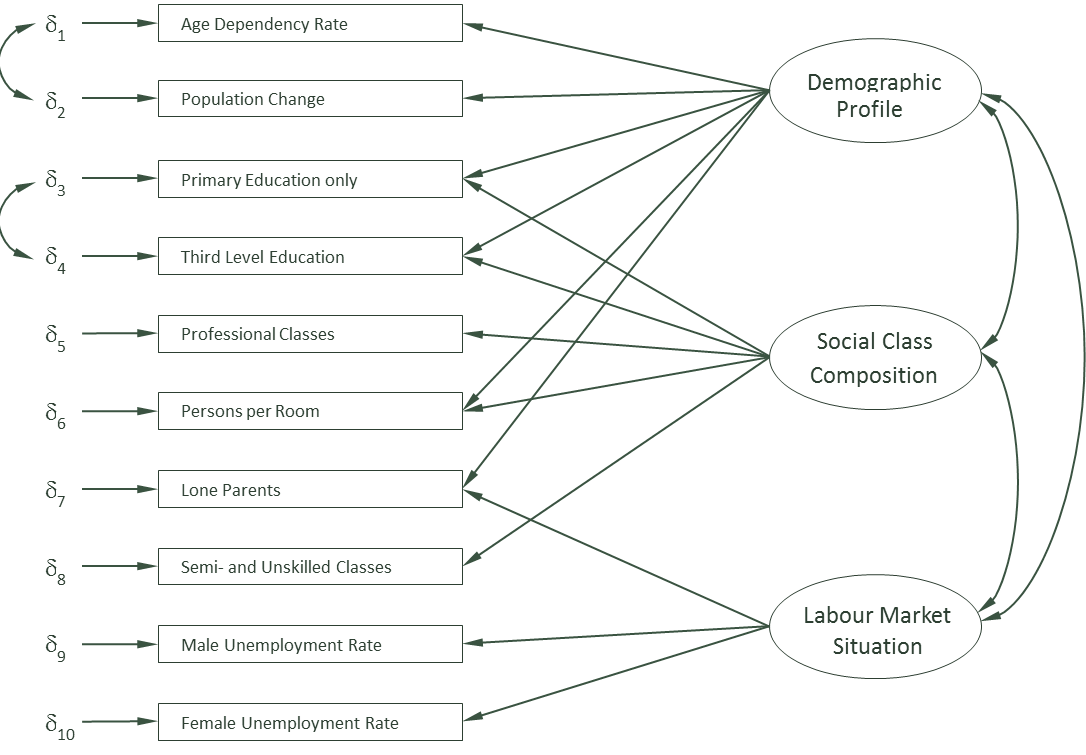
**Figure A1: The variables and associated coefficients used in the development of the HP index (Haase and Pratschke 2017 pg. 4)**

**
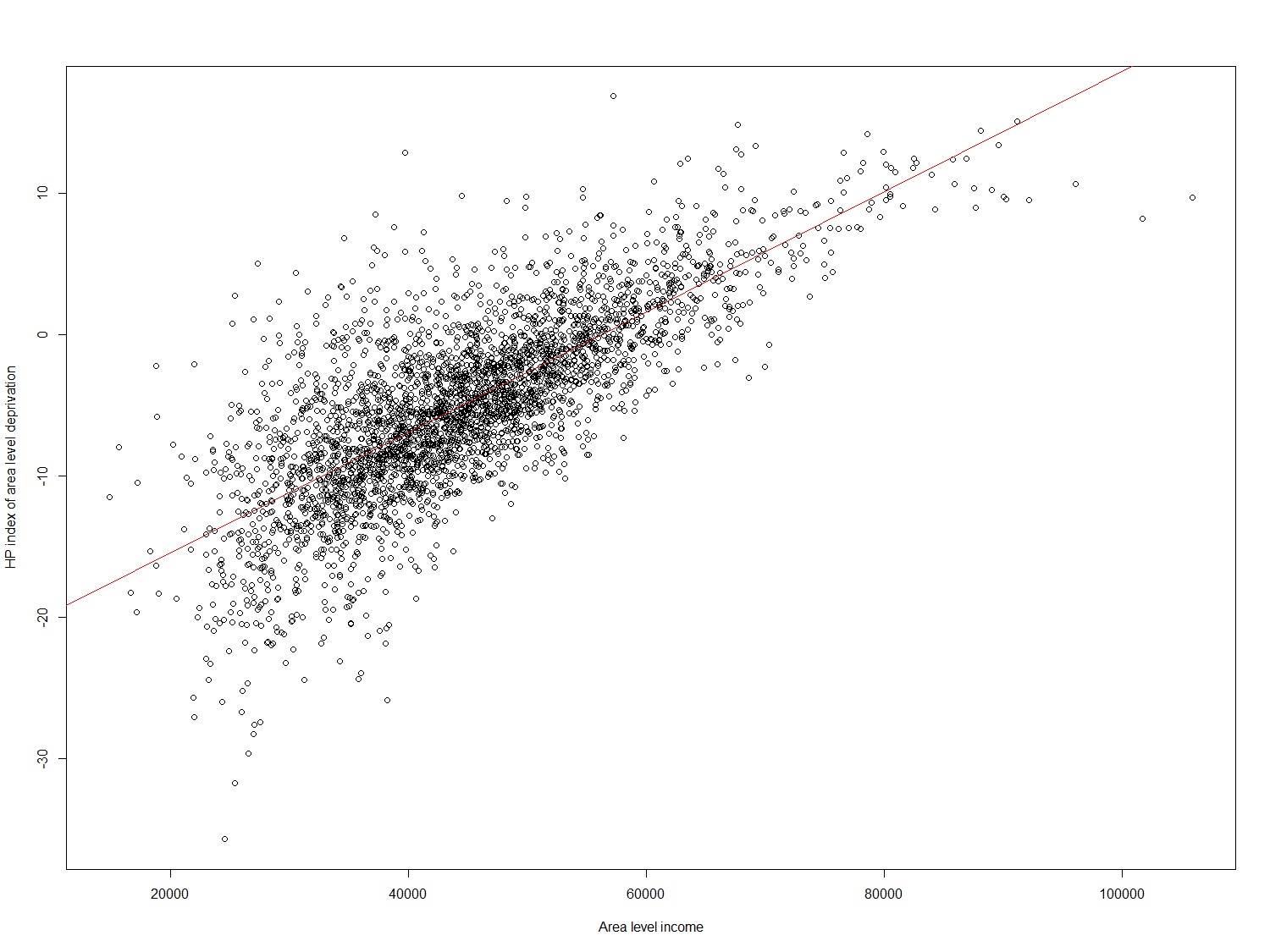
Figure A2: Graph of HP deprivation index against area-level median income by electoral district**

The following sections of this Supplementary File document additional modelling results. These models are additional analyses and sensitivity checks of the main results of the paper.

1. **Models with other measures of geographic supply**

| **GP visit in previous 4 weeks** | **Reference category** | | **Basic model** | | **Basic model with area-level deprivation** | **Full model** |
| --- | --- | --- | --- | --- | --- | --- |
| Model | |  | | (1) | (2) | (3) |
| Distance quintile 2 | | Distance quintile 1 (smallest distance) | | 1.209*  (0.113) | 1.211*  (0.114) | 1.257*  (0.126) |
| Distance quintile 3 | | Distance quintile 1 (smallest distance) | | 1.124  (0.107) | 1.126  (0.108) | 1.221  (0.126) |
| Distance quintile 4 | | Distance quintile 1 (smallest distance) | | 1.014  (0.0938) | 1.044  (0.099) | 1.173  (0.128) |
| Distance quintile 5  (furthest from GPs) | | Distance quintile 1 (smallest distance) | | 1.025  (0.0951) | 1.011  (0.098) | 1.090  (0.136) |
| Male | | Female | | 0.724***  (0.0423) | 0.723***  (0.0424) | 0.727***  (0.0456) |
| Age 25-44 | | Age 18-24 | | 0.951  (0.120) | 0.960  (0.122) | 0.867  (0.120) |
| Age 45-64 | | Age 18-24 | | 1.311*  (0.164) | 1.299*  (0.163) | 0.909  (0.126) |
| Age 65+ | | Age 18-24 | | 2.809***  (0.349) | 2.738***  (0.343) | 1.018  (0.149) |
| \| Deprivation quintile 1  (Most deprived quintile) \| \| --- \| | | **Least deprived quintile** | |  | 1.787***  (0.174) | 1.421**  (0.159) |
| Deprivation quintile 2 | | **Least deprived quintile** | |  | 1.387**  (0.139) | 1.254*  (0.139) |
| \| Deprivation quintile 3 \| \| --- \| \|  \| | | **Least deprived quintile** | |  | 1.469***  (0.149) | 1.344**  (0.148) |
| \| Deprivation quintile 4 \| \| --- \| | | **Least deprived quintile** | |  | 1.256*  (0.127) | 1.262*  (0.136) |
| \| Secondary educated \| \| --- \| | | Primary educated | |  |  | 0.926  (0.099) |
| \| Tertiary educated \| \| --- \| | | Primary educated | |  |  | 1.005  (0.122) |
| \| Married \| \| --- \| | | Unmarried | |  |  | 1.048  (0.0700) |
| \| Manual labourer \| \| --- \| | | Other profession | |  |  | 0.946  (0.084) |
| Smoker | | Non-smoker | |  |  | 0.968  (0.0841) |
| \| Private health insurance \| \| --- \| | | No private health insurance | |  |  | 1.162*  (0.0885) |
| GP visit card holder | | No medical card | |  |  | 1.636***  (0.203) |
| Medical card holder | | No medical card | |  |  | 2.098***  (0.175) |
| \| Region: Munster \| \| --- \| \|  \| | | Region:Dublin | |  |  | 0.797*  (0.0790) |
| \| Region: Rural Leinster \| \| --- \| \|  \| | | Region:Dublin | |  |  | 0.836  (0.080) |
| Region: Connaught/Ulster | | Region:Dublin | |  |  | 0.869  (0.092) |
| Urban | | Rural | |  |  | 0.961  (0.0877) |
| \| Long term illness \| \| --- \| \|  \| | | No long term illness | |  |  | 1.924***  (0.144) |
| \| Good or better self-rated health \| \| --- \| \|  \| | | Fair, poor or very bad self rated health | |  |  | 0.495***  (0.043) |
| \| Arthritis \| \| --- \| | | No arthritis | |  |  | 1.238*  (0.118) |
| Diabetes | | No diabetes | |  |  | 1.084  (0.151) |
| High blood pressure | | No high blood pressure | |  |  | 1.411***  (0.120) |
| N | |  | | 6,309 | 6,309 | 6,309 |
| Log likelihood | |  | | -3568.27 | -3548.87 | -3293.95 |
| Statistical significance indicated by * p < 0.05 ** p < 0.01 *** p < 0.001. Robust standard errors in parentheses. 95% Confidence intervals in square brackets.  Sample size differs from those reported in the main manuscript because fewer distance to GP was unobserved for some respondents | | | | | | |

**Table A2: OR, logistic regression on monthly utilisation with distance to GP as a proxy for GP supply**

| **GP visit in previous 4 weeks** | **Reference category** | **Basic model** | **Basic model with area-level deprivation** | **Full model** |  |
| --- | --- | --- | --- | --- | --- |
| Model |  | (1) | (2) | (3) |  |
| Workload quintile 1 | Workload quintile 1 (smallest distance) | 1.123  (0.102) | 1.101  (0.100) | 1.120  (0.107) |  |
| Workload quintile 2 | Workload quintile 1 (smallest distance) | 1.294**  (0.120) | 1.241*  (0.116) | 1.200  (0.119) |  |
| Workload quintile 3 | Workload quintile 1 (smallest distance) | 1.145  (0.106) | 1.110  (0.105) | 1.142  (0.115) |  |
| Workload quintile 4 | Workload quintile 1 (smallest distance) | 1.090  (0.103) | 1.068  (0.102) | 1.106  (0.111) |  |
| Male | Female | 0.722***  (0.0422) | 0.721***  (0.0422) | 0.726***  (0.046) |  |
| Age 25-44 | Age 18-24 | 0.955  (0.121) | 0.965  (0.122) | 0.871  (0.121) |  |
| Age 45-64 | Age 18-24 | 1.305*  (0.163) | 1.297*  (0.163) | 0.913  (0.126) |  |
| Age 65+ | Age 18-24 | 2.791***  (0.347) | 2.728***  (0.342) | 1.020  (0.149) |  |
| \| Deprivation quintile 1  (Most deprived quintile) \| \| --- \| | **Least deprived quintile** |  | 1.760***  (0.172) | 1.407**  (0.156) |  |
| Deprivation quintile 2 | **Least deprived quintile** |  | 1.326**  (0.130) | 1.240  (0.138) |  |
| \| Deprivation quintile 3 \| \| --- \| \|  \| | **Least deprived quintile** |  | 1.411***  (0.140) | 1.337**  (0.148) |  |
| \| Deprivation quintile 4 \| \| --- \| | **Least deprived quintile** |  | 1.230*  (0.122) | 1.263*  (0.136) |  |
| \| Secondary educated \| \| --- \| | Primary educated |  |  | 0.922  (0.099) |  |
| \| Tertiary educated \| \| --- \| | Primary educated |  |  | 1.003  (0.122) |  |
| \| Married \| \| --- \| | Unmarried |  |  | 1.052  (0.070) |  |
| \| Manual labourer \| \| --- \| | Other profession |  |  | 0.947  (0.084) |  |
| Smoker | Non-smoker |  |  | 0.963  (0.0837) |  |
| \| Private health insurance \| \| --- \| | No private health insurance |  |  | 1.163*  (0.0885) |  |
| GP visit card holder | No medical card |  |  | 1.645***  (0.204) |  |
| Medical card holder | No medical card |  |  | 2.095***  (0.175) |  |
| \| Region: Munster \| \| --- \| \|  \| | Region: Dublin |  |  | 0.804*  (0.0787) |  |
| \| Region: Rural Leinster \| \| --- \| \|  \| | Region: Dublin |  |  | 0.847  (0.080) |  |
| Region: Connaught/Ulster | Region: Dublin |  |  | 0.876  (0.0912) |  |
| Urban | Rural |  |  | 1.012  (0.0747) |  |
| \| Long term illness \| \| --- \| \|  \| | No long term illness |  |  | 1.916***  (0.144) ) |  |
| \| Good or better self-rated health \| \| --- \| \|  \| | Fair, poor or very bad self rated health |  |  | 0.498***  (0.0435) |  |
| \| Arthritis \| \| --- \| | No arthritis |  |  | 1.243*  (0.118) |  |
| Diabetes | No diabetes |  |  | 1.071  (0.150) |  |
| High blood pressure | No high blood pressure |  |  | 1.411***  (0.120) |  |
| N |  | 6,309 | 6,309 | 6,309 |  |
| Log likelihood |  | -3567.35 | -3549.01 | -3295.43 |  |
| Statistical significance indicated by * p < 0.05 ** p < 0.01 *** p < 0.001. Robust standard errors in parentheses. 95% Confidence intervals in square brackets.  Sample size differs from those reported in the main manuscript because GP workload was unobserved for some respondents | | | | |  |

**Table A3: OR for logistic regression model on monthly utilisation with GP workload as a proxy for GP supply**

| **GP visit in previous 4 weeks** | **Reference category** | **Basic model** | | **Basic model with area-level deprivation** | **Full model**  **including**  **GP supply** |
| --- | --- | --- | --- | --- | --- |
| Model |  | (1) | | (2) | (3) |
| \| GP concentration quintile 1  (Lowest supply of GPs in locality) \| \| --- \| | *Zero GPs in walking distance* | | 1.055  (0.094) | 1.023  (0.0926) | 0.980  (0.0978) |
| GP concentration quintile 2 | *Zero GPs in walking distance* | | 1.274*  (0.120) | 1.303**  (0.127) | 1.212  (0.157) |
| \| GP concentration quintile 3 \| \| --- \| \|  \| | *Zero GPs in walking distance* | | 1.051  (0.104) | 1.056  (0.106) | 0.960  (0.129) |
| \| GP concentration quintile 4 \| \| --- \| | *Zero GPs in walking distance* | | 1.018  (0.0955) | 1.020  (0.0994) | 0.871  (0.122) |
| GP concentration quintile 5  (Most GPs in locality) | *Zero GPs in walking distance* | | 1.116  (0.109) | 1.161  (0.119) | 1.017  (0.150) |
| Male | Female | | 0.724***  (0.0422) | 0.723***  (0.0423) | 0.725***  (0.045) |
| Age 25-44 | Age 18-24 | | 0.954  (0.120) | 0.963  (0.122) | 0.867  (0.120) |
| Age 45-64 | Age 18-24 | | 1.323*  (0.165) | 1.311*  (0.164) | 0.910  (0.125) |
| Age 65+ | Age 18-24 | | 2.829***  (0.351) | 2.755***  (0.344) | 1.022  (0.149) |
| \| Deprivation quintile 1  (Most deprived quintile) \| \| --- \| | **Least deprived quintile** | |  | 1.800***  (0.176) | 1.430**  (0.159) |
| Deprivation quintile 2 | **Least deprived quintile** | |  | 1.411***  (0.142) | 1.260*  (0.140) |
| \| Deprivation quintile 3 \| \| --- \| \|  \| | **Least deprived quintile** | |  | 1.484***  (0.152) | 1.342**  (0.148) |
| \| Deprivation quintile 4 \| \| --- \| | **Least deprived quintile** | |  | 1.259*  (0.128) | 1.255*  (0.135) |
| \| Secondary educated \| \| --- \| | Primary educated | |  |  | 0.928  (0.100) |
| \| Tertiary educated \| \| --- \| | Primary educated | |  |  | 1.004  (0.122) |
| \| Married \| \| --- \| | Unmarried | |  |  | 1.056  (0.0704) |
| \| Manual labourer \| \| --- \| | Other profession | |  |  | 0.943  (0.0836) |
| Smoker | Non-smoker | |  |  | 0.976  (0.0843) |
| \| Private health insurance \| \| --- \| | No private health insurance | |  |  | 1.160  (0.0881) |
| GP visit card holder | No medical card | |  |  | 1.649***  (0.206) |
| Medical card holder | No medical card | |  |  | 2.100***  (0.175) |
| \| Region: Munster \| \| --- \| \|  \| | Region: Dublin | |  |  | 0.794*  (0.0850) |
| \| Region: Rural Leinster \| \| --- \| \|  \| | Region: Dublin | |  |  | 0.814*  (0.083) |
| Region: Connaught/Ulster | Region: Dublin | |  |  | 0.845  (0.0963) |
| Urban | Rural | |  |  | 0.987  (0.100) |
| \| Long term illness \| \| --- \| \|  \| | No long term illness | |  |  | 1.920***  (0.144) |
| \| Good or better self-rated health \| \| --- \| \|  \| | Fair, poor or very bad self rated health | |  |  | 0.496***  (0.0433) |
| \| Arthritis \| \| --- \| | No arthritis | |  |  | 1.241*  (0.118) |
| Diabetes | No diabetes | |  |  | 1.068  (0.149) |
| High blood pressure | No high blood pressure | |  |  | 1.415***  (0.120) |
| N |  | 6,309 | | 6,309 | 6,309 |
| Log likelihood |  | -3579.74 | | -3559.01 | -3303.97 |
| Statistical significance indicated by * p < 0.05 ** p < 0.01 *** p < 0.001. Robust standard errors in parentheses. 95% Confidence intervals in square brackets. | | | | | |

**Table A4: OR, logistic regression on monthly utilisation with GP concentration as a proxy for GP supply**

1. **Alternative models and sensitivity checks**

Besides the logistic regression used in this analysis, further models were explored to provide robustness checks for the main results. A logistic regression on whether an individual had visited the GP in the past year and an ordinary least squares regression on the number of GP visits in the past year were also carried out. The logistic regression suggests little increase in the likelihood of an individual in a more deprived area attending the GP in the previous 12 months but the OLS regression indicated an increase in frequency of utilisation in the previous year. These results broadly concur with the findings of the logistic regression.

*Logistic Regression on use of GP in the past year*

**Table A5: OR from logistic regression model of whether an individual had visited the GP in the previous 12 months**

| OR on having gone to the GP in the past year | |  |  |
| --- | --- | --- | --- |
| **Reference category: least deprived quintile (quintile 5)** | Model with age and sex | Full model w/o Supply | Full model |
| Deprivation quintile 1  (Most deprived) | 1.398***  (0.140) | 1.220  (0.137) | 1.211  (0.137) |
| Deprivation quintile 2 | 1.074  (0.100) | 0.983  (0.103) | 0.980  (0.103) |
| Deprivation quintile 3 | 1.096  (0.104) | 1.006  (0.106) | 1.005  (0.106) |
| Deprivation quintile 4 | 1.031  (0.097) | 0.982  (0.098) | 0.983  (0.098) |
| GP concentration quintile 1  (Lowest supply of GPs in locality) |  |  | -0.174  (0.103) |
| GP concentration quintile 2 |  |  | -0.068  (0.130) |
| GP concentration quintile 3 |  |  | -0.070  (0.137) |
| GP concentration quintile 4 |  |  | 0.087  (0.143) |
| GP concentration quintile 5  (Most GPs in locality) |  |  | 0.040  (0.151) |
| Log likelihood | -3270.22 | -3015.46 | -3014.40 |
| Number of observations | 6326 | 6326 | 6326 |
| Robust standard errors in parentheses  * p < 0.05 ** p < 0.01 *** p < 0.001  Full model includes: medical card status, marital status, unskilled labourer, region, level of education, urbanity, Illness in past 12 months, Diabetes, Arthritis. | | | |

*Ordinary Least Squared Regression on use of GP in the past 4 weeks*

**Table A6: Ordinary least squares regression on number of visitations to the GPs in the previous 4 weeks**

| Number of GP visits in the past month | |  |  |
| --- | --- | --- | --- |
| **Reference category: least deprived quintile (quintile 5)** | Model with age and sex | Full model w/o Supply | Full model |
| Deprivation quintile 1  (Most deprived) | 2.420***  (0.371) | 1.459  (0.384) | 1.499***  (0.389) |
| Deprivation quintile 2 | 0.859**  (0.310) | -0.003  (0.333) | 0.574  (0.334) |
| Deprivation quintile 3 | 1.015**  (0.315) | 0.001  (0.326) | 0.713*  (0.328) |
| Deprivation quintile 4 | 0.005  (0.302) | 0.603*  (0.306) | 0.613*  (0.307) |
| GP concentration quintile 1  (Lowest supply of GPs in locality) |  |  | -0.008  (0.025) |
| GP concentration quintile 2 |  |  | 0.035  (0.032) |
| GP concentration quintile 3 |  |  | -0.017  (0.032) |
| GP concentration quintile 4 |  |  | 0.011  (0.036) |
| GP concentration quintile 5  (Most GPs in locality) |  |  | -0.002  (0.036) |
| Number of observations | 6326 | 6326 | 6326 |
| Robust standard errors in parentheses  * p < 0.05 ** p < 0.01 *** p < 0.001  Full model includes: medical card status, marital status, unskilled labourer, region, level of education, urbanity, Illness in past 12 months, Diabetes, Arthritis. | | | |

**Figure A3
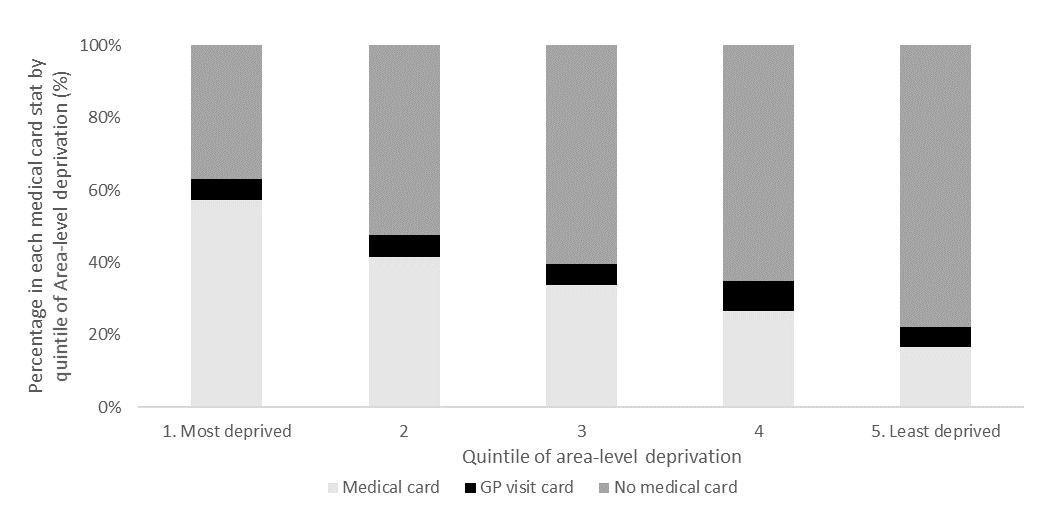
: Description of medical card status in sample by quintile of area-level deprivation**

**References**

Haase, T., and Pratschke, J. 2017. ‘The 2016 Pobal HP Deprivation Index for Small Areas (SA): Introduction and Reference Tables’.
